# Supplementary figures and images for: Human Pluripotent Stem Cell-Derived Neural Progenitor Cells Promote Retinal Ganglion Cell Survival and Axon Recovery in an Optic Nerve Compression Animal Model
Source: Int J Mol Sci. 2021 Nov 20;22(22):12529. doi: 10.3390/ijms222212529 (PMC8622638; doi:10.3390/ijms222212529)

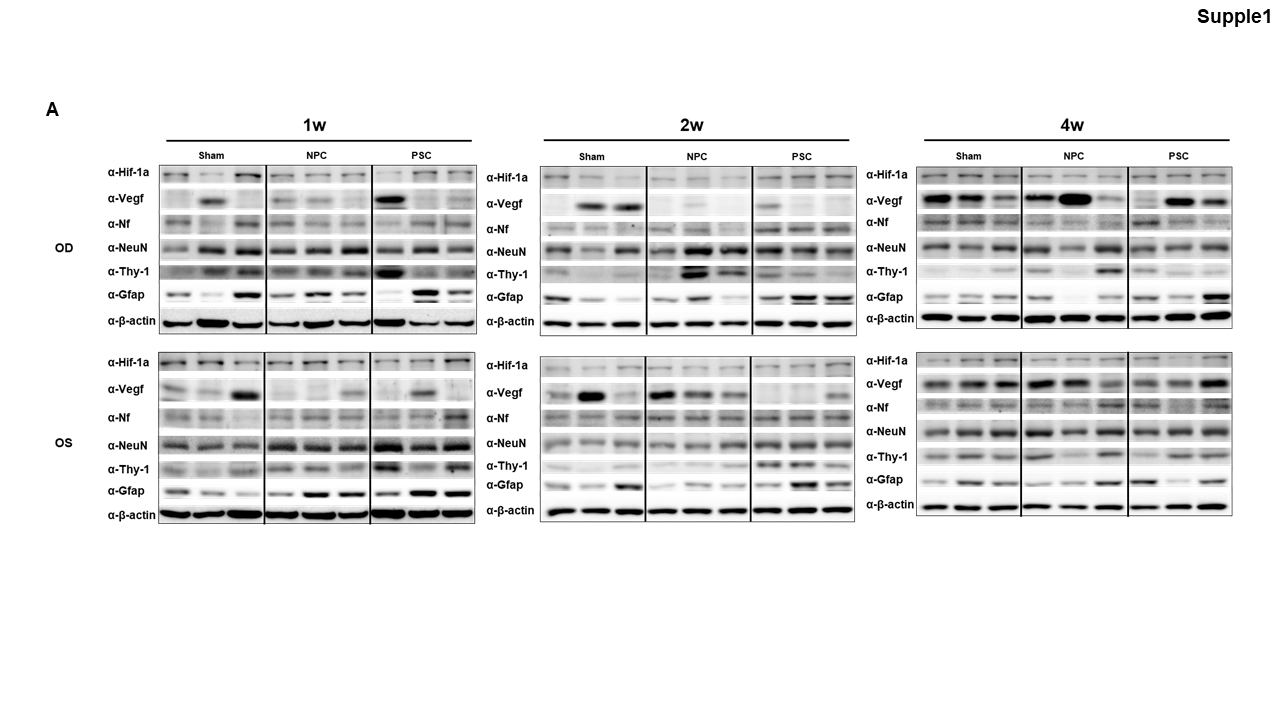

Supplement: Supplementary file 1 [file ijms-22-12529-s001.zip › Supple Figure 1A.TIF]

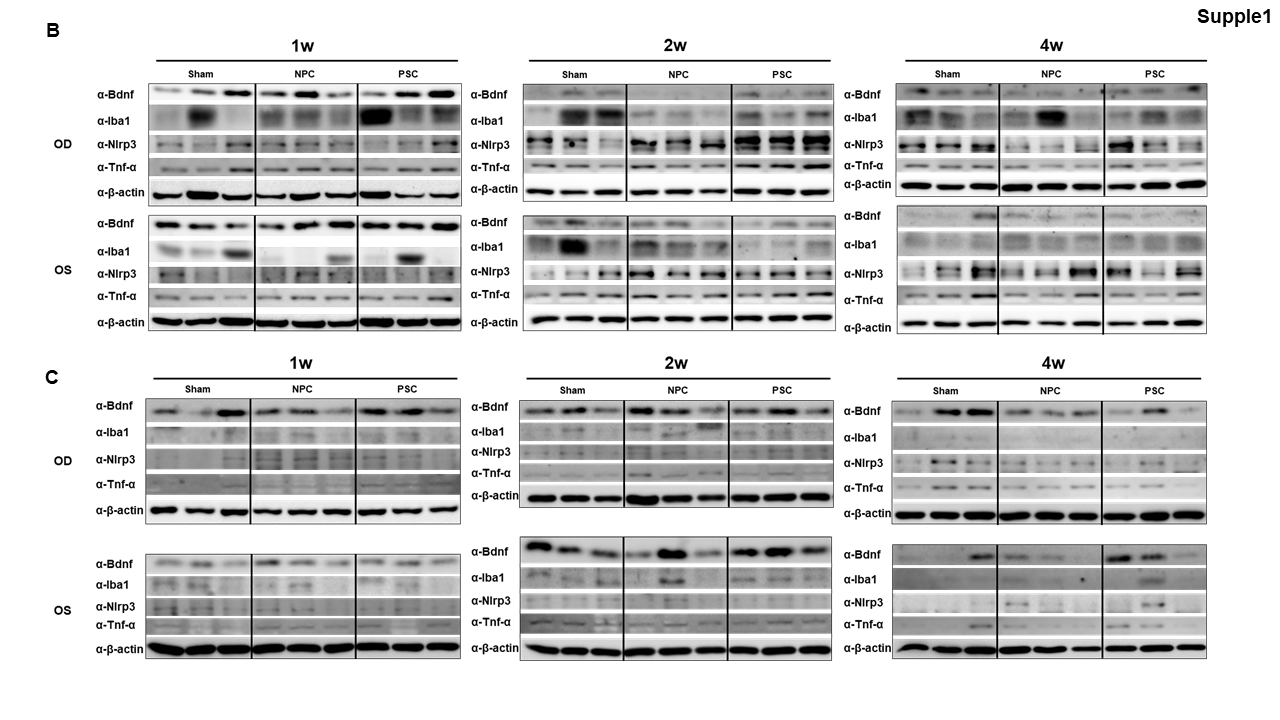

Supplement: Supplementary file 1 [file ijms-22-12529-s001.zip › Supple Figure 1BC.TIF]
